# Development and Application of Attenuated Plant Viruses as Biological Control Agents in Japan

**DOI:** 10.3390/v16040517

**Published:** 2024-03-27

**Authors:** Yasuhiro Tomitaka, Yoshifumi Shimomoto, Bo-Song Ryang, Kazusa Hayashi, Tomoka Oki, Momoko Matsuyama, Ken-Taro Sekine

**Affiliations:** 1 Institute for Plant Protection, National Agricultural Research Organization (NARO), 2-1-18, Kannondai, Tsukuba 305-8666, Japan; matsuyamam965@affrc.go.jp; 2 Kochi Agricultural Research Center, 1100 Hataeda, Nankoku 783-0023, Japan; yoshifumi_shimomoto@ken2.pref.kochi.lg.jp (Y.S.); kazusa_hayashi@ken3.pref.kochi.lg.jp (K.H.); tomoka_oki@ken3.pref.kochi.lg.jp (T.O.); 3 Kyoto Biken Laboratories, Inc., 16 Nijushi, Makishima, Uji 611-0041, Japan; ryo@kyotobiken.co.jp; 4 Faculty of Agriculture, University of the Ryukyus, 1 Senbaru, Nakagashiragun, Nishihara 611-0041, Japan; k-sekine@agr.u-ryukyu.ac.jp

**Keywords:** cross-protection, attenuated strains, plant vaccine

## Abstract

In 1929, it was reported that yellowing symptoms caused by a tobacco mosaic virus (TMV) yellow mosaic isolate were suppressed in tobacco plants that were systemically infected with a TMV light green isolate. Similar to vaccination, the phenomenon of cross-protection involves a whole plant being infected with an attenuated virus and involves the same or a closely related virus species. Therefore, attenuated viruses function as biological control agents. In Japan, many studies have been performed on cross-protection. For example, the tomato mosaic virus (ToMV)-L_11_A strain is an attenuated isolate developed by researchers and shows high control efficiency against wild-type ToMV in commercial tomato crops. Recently, an attenuated isolate of zucchini yellow mosaic virus (ZYMV)-2002 was developed and registered as a biological pesticide to control cucumber mosaic disease. In addition, attenuated isolates of pepper mild mottle virus (PMMoV), cucumber mosaic virus (CMV), tobacco mild green mosaic virus (TMGMV), melon yellow spot virus (MYSV), and watermelon mosaic virus (WMV) have been developed in Japan. These attenuated viruses, sometimes called plant vaccines, can be used not only as single vaccines but also as multiple vaccines. In this review, we provide an overview of studies on attenuated plant viruses developed in Japan. We also discuss the application of the attenuated strains, including the production of vaccinated seedlings.

## 1. Introduction

Plant viruses cause significant economic losses by reducing crop yield and quality. Many plant viruses are transmitted by insect vectors, such as aphids, thrips, and whiteflies. However, since pesticides against plant viruses do not exist, controlling viral vectors using chemical insecticides is an important measure often used to control plant viruses. The recent development of insecticide resistance in some insects has resulted in increased crop damage. Therefore, it is becoming necessary to develop novel control methods that do not involve insecticides, such as resistance genes, which can be generally effective in inhibiting the infection of plant viruses. Accordingly, resistance genes for several plant viruses have been introduced into major crops such as tomato and green pepper. However, the emergence of resistance-breaking isolates is a problem. In addition, no resistance genes against plant viruses in minor crops such as Chinese lantern plant have yet been found. For these plants, the development of attenuated viruses is a critical tool for controlling viral disease.

Cross-protection protects plants from secondary infection by other viral isolates. The cross-protection effect was first demonstrated by McKinney [1], who observed that the appearance of yellow symptoms caused by a tobacco mosaic virus (TMV) yellow mosaic isolate was suppressed in tobacco plants that were systemically infected with a TMV light green isolate. In contrast, the TMV mild dark green isolate did not result in the suppression of yellow symptoms. Cross-protection has been demonstrated in numerous viruses, including potato virus X, potato leafroll virus, and citrus tristeza virus (CTV) [2,3,4,5]. Once an attenuated virus is introduced and infects an entire plant, it cannot infect the same or closely related virus species. Thus, attenuated viruses served as important biological control agents.

In 1933, Salaman first investigated the cross-protection effects of plant viruses and showed that a mild isolate of potato virus X (PVX) inhibited the infection of a severe PVX isolate [4]. Later, Holmes developed a mild isolate of TMV by heat treating a virulent isolate of TMV and reported that the symptoms caused by infection with the virulent isolate were suppressed in plants infected with the mild one [6]. In 1951, a mild isolate of CTV was developed for field tests in which more than 2000 citrus rootstocks were used [7]. These results indicated that citrus plants infected with mild isolates of CTV were protected against more severe isolates of CTV. A similar study design was used by Posnette and Todd, who conducted field tests on a mild isolate of cacao virus 1A in an African field where swollen shoot disease was prevalent [8]. In that study, 273 out of 387 uninfected trees showed severe symptoms of infection, whereas only 35 of 416 trees that had been infected with the mild isolate showed severe symptoms. This result was taken as evidence of successful practical protection of cacao trees by inoculation with a mild viral isolate. Thus, both basic and practical research on attenuated viruses has been conducted for many years.

Lecoq listed the following characteristics to develop attenuated isolates for field cross-protection [9]: (1) mild symptoms, adequate marketable yield, and crop quality; (2) high histocompatibility with inoculated plants; (3) genetic stability; (4) limited unintentional spread; (5) protection against a wide range of wild-type isolates; and (6) effective control and ease of application. Each of these characteristics remain important for the field use of attenuated isolates.

Many attenuated viruses have been developed for practical use worldwide [9,10,11]. Of these, one of the most famous is pepino mosaic virus (PepMV), which causes serious economic loss in tomato crops [12]. The use of attenuated isolates has been considered against virulent PepMV, and mild isolates such as 1906, VX1, and VC1 have been developed for practical use [13,14,15]. For example, a PepMV vaccine derived from the 1906 isolate, known as PMV-01, is commercially available in 23 different countries in North America and Europe (https://dcm-info.com/int/en/innovations/pmv-01-the-biological-control-agent-against-pepmv; accessed on 19 March 2024). More recently, the use of a combination of two mild isolates, Sp13 and SP5, sourced from the EU and CH2 strains, respectively, has been proposed for controlling disease caused by virulent PepMV [16,17]. In 2021, 117 million tomato plants planted on ca. 14,700 ha were inoculated with the two mild isolates [17].

There are many studies on attenuated isolates and the cross-protection of plant viruses that are specifically localized to Japan [18]. In particular, recent progress in the use of these isolates has resulted in increased dissemination of attenuated viruses. In this review, we provide an overview of recent studies on attenuated plant virus strains developed in Japan. We also discuss the application of the attenuated viruses, including the production of vaccinated seedlings.

## 2. History of the Development of Attenuated Viruses in Japan

Japan has a long history of the development of attenuated viruses, and many studies on viral cross-protection have been reported [19,20,21,22]. In particular, Japanese researchers have developed attenuated viruses belonging to the genera *Closterovirus* [23,24], *Cucumovirus* [25,26,27,28], *Potyvirus* [29,30,31,32,33,34], *Tobamovirus* [35,36,37,38,39,40,41,42,43,44], and *Tospovirus* [45] and assessed their cross-protective effects (Table 1). Traditionally, attenuated viruses have been isolated from naturally occurring mutants or via mutagenesis of wild-type viruses using nitrous acid or ultraviolet (UV) irradiation. Sodium nitrite is a well-known mutagen that deaminates cytosine and adenine to produce uracil and hypoxanthine, while UV irradiation has been used to isolate attenuated viruses that form pyrimidine dimers in DNA and RNA [22,46]. In addition, viruses have been isolated from plants incubated at both low and high temperatures (Table 1). Overall, sodium nitrite and low-temperature treatments are often used together due to their high efficiency in inducing mutations.

CTV, which belongs to the genus *Closterovirus*, is important for fruit tree production, since many citrus cultivars are infected with wild-type CTV [23,24]. However, when virus-free seedlings are planted in the field, the insect vectors *Toxoptera citricida* and *Aphis gossypii*, which are also common in citrus orchards, quickly transmit wild-type CTV to these new plants, causing severe damage. To address this problem, a mild isolate of CTV known as M-16A was used to control wild-type CTV and related symptoms. In this line of research, the cross-protective effect against severe isolates of CTV was investigated for about eight years. The results showed that the yields of trees inoculated with mild isolates increased by ca. 50% [23]. Moreover, the size of fruit harvested from inoculated plants was larger than those of plants infected with wild-type CTV.

Tomato mosaic virus (ToMV)-L_11_A isolate is a well-known attenuated virus developed using these treatments and is known to provide a high degree of protection against wild-type ToMV strains in commercial tomato (*Solanum lycopersicum* L.) crops [36]. Decades ago, this isolate was widely used throughout Japan. For example, in Chiba Prefecture, ca. 20% of all tomato farmers had introduced ToMV-L_11_A before new Tobamovirus-resistant tomato varieties, such as *Tm-2* and *Tm-2^a^* had been developed [47]. Moreover, when tomato plants were inoculated with ToMV-L_11_A, the fruit yield was found to be higher than that of an uninoculated control [48,49]. Sequencing analyses identified ten nucleotide substitutions between the complete nucleotide sequences of ToMV-L and -L_11_A [50]. Among them, a substitution in the replication protein (nucleotide _1117_G > A: amino acid _349_Cys > Tyr) is likely responsible for symptom attenuation. Moreover, another attenuated ToMV has been isolated from ToMV-L_11_A and an attenuated isolate, ToMV-L_11_A237, has been derived from ToMV-L_11_A [44].

Cucumber green mottle mosaic virus (CGMMV) is also a well-known crop virus in Japan. An attenuated isolate known as CGMMV-SH33b has been developed by combining high-temperature, nitrite, and UV irradiation treatments [35]. Although the attenuated virus causes mild symptoms in leaves, it does not generate symptoms in melon fruit. Furthermore, comparisons of the nucleotide sequence between CGMMV-SH33b and the virulent isolate identified nine substitutions. Of these, three nucleotide substitutions that resulted in changes in amino acid sequences were identified within the replication protein gene. Impaired siRNA binding activity plays an important role in the attenuation of CGMMV-SH33b, which involves mutations in _480_Glu > Gly, _1124_Ala > Val, _1157_Asn > Asp, and _1397_Pro > Ser in replication proteins [51].

In Japan, attenuated cucumber mosaic virus (CMV) isolates have been developed via selecting natural variants, manipulating the RNA genome, and by the addition of satellite RNA [25,26,27,28]. For example, CMV-K02 has been developed to inoculate against mosaic disease of tomatoes [27]. It possesses a satellite RNA containing 368 nucleotides that was involved in attenuation. Field tests revealed that tomato plants treated with CMV-K02 had a 20–200% higher yield than non-treated plants [27], leading growers to purchase tomato seedlings inoculated with the attenuated CMV isolate throughout the country. Moreover, attenuated isolate of CMV is also used to control the disease in the Japanese gentian (*Gentiana scabra*) [52].

In addition, three attenuated isolates of pepper mild mottle virus (PMMoV) (i.e., C-1421, Pa18, and TPO-2-19) have been independently obtained by heat treatment and artificial selection [37,38,39].

As described above, advancement in the development and analysis of isolates has been accompanied by complementary advances in the development and application of new attenuated viruses. In what follows, we discuss attenuated viruses recently developed in Japan.

### 2.1. Attenuated Virus Strains for Mosaic Disease of Cucumber

**Figure 1:**
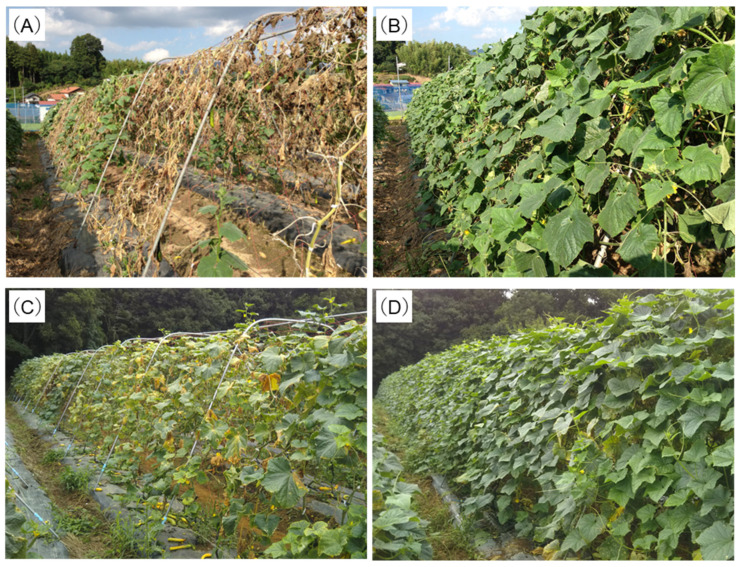
Comparison of symptoms observed in untreated and attenuated strain-inoculated cucumber plants during a field experiment. Panels (**A**,**B**) show the symptoms of untreated and ZYMV-2002-inoculated plants, respectively. Untreated plants exhibited mosaic and stunting symptoms, while inoculated plants remained asymptomatic. Panels (**C**,**D**) depict symptoms of untreated and CMV- and WMV-attenuated isolate-inoculated cucumbers, respectively. Here, uninoculated plants exhibited mosaic, wilting, and yellowing symptoms, whereas inoculated plants exhibited mild symptoms.

Cucumber is an important crop, but viral diseases transmitted by insects such as aphids have become a major problem for cucumber growers. Cucumbers are commonly affected by zucchini yellow mosaic virus (ZYMV) from the summer to early autumn. ZYMV belongs to the genus *Potyvirus*, and causes serious damage to cucumber (*Cucumis sativus* L.) in Japan and throughout the world. Plants infected with ZYMV show severe leaf and fruit malformation and wilting symptoms [53]. An attenuated virus named ZYMV-2002 has recently been obtained via heat treatment. The ZYMV-2002 isolate has been found to cause only very mild or no symptoms at all on host cucumber plants. Furthermore, inoculated cucumber plants showed levels of fruit productivity that were comparable to healthy control plants under field conditions. In field experiments, vaccinated plants significantly suppressed infection by wild-type ZYMV, disease progression, and reduction in fruit yield and quality, even when other viruses were also present [33] (Figure 1A,B). Currently, the attenuated ZYMV-2002 isolate is the most widely used in cucumber, and was the first attenuated virus registered in Japan, where it is marketed under the trade name Cubio ZY-02 (Ministry of Agriculture, Forestry and Fisheries Registration No. 22152, Kyoto Biken Laboratories, Inc., Kyoto, Japan).

CMV and watermelon mosaic virus (WMV) are two other important viruses affecting cucurbits. CMV causes mosaic disease in the leaves and decreases fruit yield, and in some cases, also lowers fruit quality and generates yellow spot symptoms. WMV, which often coexists with CMV, intensifies CMV symptoms via a synergistic effect. Attenuated CMV and WMV isolates have recently been developed to control these diseases [54]. In one study, CMV and WMV isolates were co-inoculated into cucumber plants, which were planted in an open field where they were exposed to wild-type viruses. The authors found that attenuated isolates of CMV and WMV inhibited symptoms of both CMV and WMV infection (Figs. 1C and 1D). Moreover, the growth inhibition rate of plants inoculated with CMV and WMV isolates decreased, whereas the marketable fruit rate increased.

ZYMV, CMV, and WMV are generally transmitted by aphids in a non-persistent manner. Interestingly, none of the attenuated isolates of the viruses were found to be transmitted by aphids [33]. This is important because it minimizes the environmental impact of attenuated viruses.

### 2.2. Attenuated Virus Strains Inhibit Mosaic Disease of Green Pepper

Green peppers are another important crop in Japan, and mosaic disease caused by tobamovirus infection is a major problem for green pepper cultivation. For example, pepper mild mottle virus (PMMoV), which belongs to the genus *Tobamovirus*, causes serious damage to green pepper (*Capsicum annuum* L.), and is transmitted both by seed and contact. In the field, PMMoV is first introduced via contaminated seed, and then moves from plant to plant following pruning. PMMoV infection has been found to reduce plant growth and induces leaf mosaic symptoms and fruit malformation [55], symptoms that affect marketability. Thus, in Japan, green pepper fruits are generally classified into three categories: class A (excellent), class B (good), and unsalable (unmarketable). Many malformed fruits are produced by PMMoV-infected plants, resulting in reduced revenue. At first, the soil fumigant methyl bromide was commonly used to control mosaic disease caused by PMMoV [56]. However, in 1992 this compound was deemed an ozone-depleting substance at the Fourth Meeting of the Conference of the Parties to the Montreal Protocol on Substances that Deplete the Ozone Layer. Thereafter, in most developed countries, methyl bromide has not been used since 2005, except for critical and quarantine cases. However, Japanese growers have continued to use methyl bromide for soil fumigation, but Japan agreed to phase out its use by the end of 2012 [57].

**Figure 2:**
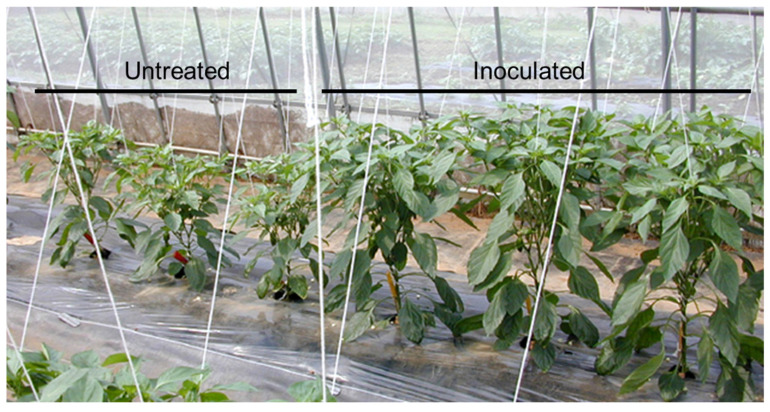
Symptoms of untreated and L3-163 attenuated isolate-inoculated green pepper plants. The virulent strain of pepper mild mottle virus was inoculated via pruning shears. Untreated plants exhibited mosaic and stunting symptoms (**left**), while plants inoculated with the isolate remained asymptomatic (**right**).

As a result, alternative methods of controlling PMMoV infection in green pepper were required, prompting greater interest in the development of attenuated PMMoV viruses. Interestingly, some wild species of *Capsicum annuum* have a Tobamovirus resistance gene known as *L*, which has four distinct alleles, *L*^1^, *L*^2^, *L*^3^, and *L*^4^ [58]. In addition, PMMoV has a pathogenic type P that is able to counter the effects of the L gene. For instance, pathotype P_1,2,3_ forms of PMMoV can overcome the *L*^1^, *L*^2^, and *L*^3^ genes, respectively [59]. Many cultivars of green pepper grown in the field have the *L*^3^ gene. However, resistance breaking isolates of the wild-type PMMoV (i.e., P_1, 2, 3_) are often found in Japanese cultivated peppers [60,61]. Therefore, an attenuated isolate known as L3-163 (P_1, 2, 3_) was developed using a heat treatment, and this was trialed on green pepper cultivars that carried the *L^3^* resistance gene [40,62]. Specifically, the cross-protection effects of the L3-163 isolate against infection by a PMMoV-virulent strain in greenhouse-cultivated green pepper plants were evaluated in the field. Plants inoculated with the attenuated isolate were found to be completely protected from infection by the virulent PMMoV strain. In these experiments, untreated green peppers showed significant mosaic symptoms, whereas plants inoculated with L3-163 did not (Figure 2). These results therefore demonstrate that the L3-163 isolate is useful as a biological control agent for green pepper cultivars grown in Japan. Although an attenuated isolate of PMMoV has been registered as a biotic pesticide, this registration has now expired.

### 2.3. Attenuated Virus Strains for Mosaic and Necrotic Disease of Chinese Lantern Plants

There has been significant interest in using attenuated isolates to inoculate against mosaic and necrotic diseases in Chinese lantern plants, *Physalis alkekengi* L. var. *franchetii.* This species, commonly known simply as the Chinese lantern plant, belongs to the *Solanaceae* family and is a traditional ornamental plant in Japan. While the cultivation of Chinese lantern plants can involve either seed or vegetative (root) propagation, the latter has become more prevalent since it preserves the desired characteristics [41]. However, the recent emergence of diseases has resulted in significant damage to many plants, including mosaic and necrotic symptoms on their leaves and calyx. Yoneda et al. conducted research on the pathogens responsible for these symptoms and determined that they were caused by infection of tobacco mild green mosaic virus (TMGMV) and/or ToMV [63]. TMGMV and ToMV, both members of the genus *Tobamovirus*, commonly affect Solanaceous plants such as tomato, green pepper, and hot pepper.

An attenuated strain of TMGMV (TMGMV-No. 4) and ToMV-L_11_A is currently used to manage mosaic and necrotic diseases in Chinese lantern plants. For example, in one study, the efficacy of single infection with TMGMV-No. 4 or ToMV-L_11_A in fields contaminated with virulent strains of both TMGMV and ToMV was assessed to determine the influence of cross-protective effects. In addition, the cross-protective effects of co-infections with TMGMV-No. 4 and ToMV-L_11_A were also evaluated. The results of this study demonstrated that cross-protective effects were higher in seedlings inoculated with both TMGMV-No. 4 and ToMV-L_11_A relative to those infected with only one of the two viruses, thereby underscoring the importance of inoculation with a cocktail of attenuated isolates for controlling viral diseases that result from multiple infections (Table 2) [42].

Moreover, since Chinese lantern plants are commonly propagated via their roots, once one Chinese lantern plant is infected with the attenuated virus, the virus can be transferred to the next generation of plants through inoculated roots, thereby reducing the time, cost, and labor associated with inoculation of TMGMV-No. 4 and ToMV-L_11_A.

**Figure 3:**
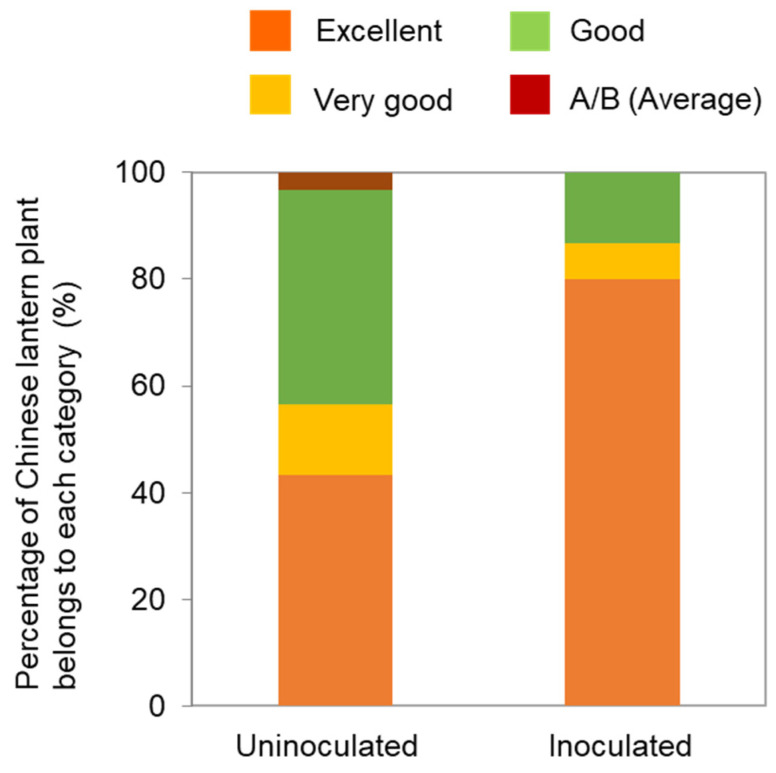
Quality comparison of uninoculated and inoculated Chinese lantern plants. The quality divided into four categories: excellent, very good, good, A/B (average), unmarketable. The unmarketable category was not shown for this experiment. Data modified from Yoneda et al. [42].

In Japan, the quality of Chinese lantern plants is categorized into five levels: excellent, very good, good, A/B (average), and unmarketable. Notably, the quality of Chinese lantern plants was higher in co-inoculated plants than in uninoculated ones (Figure 3). In particular, the number of plants in the excellent and very good categories increased in plants that had been co-inoculated with both TMGMV-No. 4 and ToMV-L_11_A. Although the mechanism underlying the attenuation of TMGMV-No. 4, including the specific nucleotide substitution responsible, remains unknown, both attenuated isolates have been effectively deployed in the field.

### 2.4. Attenuated Viral Strains against Cucumber Spotted Wilt Disease

Spotted wilt disease is highly prevalent in cucumber plants. In general, it poses a significant challenge in Japan [64,65,66] and remains a potential threat in other East Asian countries [67]. This disease is caused by infection of the melon yellow spot virus (MYSV), a member of the genus *Orthotospovirus*. It is transmitted by melon thrips (*Thrips palmi*), which has developed resistance to several insecticides, thereby exacerbating this issue. Consequently, both thrip feeding damage and MYSV infection remain serious concerns.

**Figure 4:**
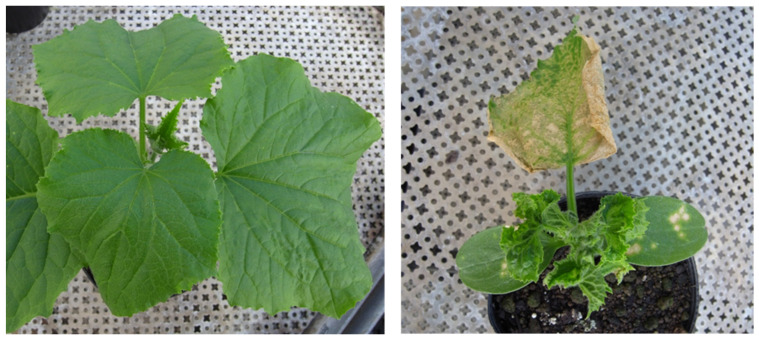
Symptoms of cucumber plants inoculated with melon yellow spot virus (MYSV). The **left** panel shows a cucumber plant inoculated with an attenuated isolate of MYSV (SA08-8), whereas the **right** panel shows a plant inoculated with a MYSV-virulent isolate (C05T). Both pictures were taken 20 days-post-inoculation.

To combat spotted wilt disease, an attenuated isolate of MYSV named SA08-8 was recently developed using heat treatment (Figure 4) [45]. In that study, the cross-protection effects of SA08-8 against MYSV-virulent strain infection transmitted via melon thrips were evaluated in greenhouse-cultivated cucumber plants. Disease severity was assessed using a disease index, where 0 represented no or mild symptoms, and 100 indicated severe mosaic symptoms, necrotic spots, or vein necrosis. The average disease indices for mock and SA08-8-inoculated plants were 63.9 and 2.7, respectively. These findings suggest that SA08-8 provides cross-protection efficiency against virulent MYSV isolates in cucumber plants for up to 56 days after the release of viruliferous thrips under greenhouse conditions [45].

Comparing the nucleotide sequences of the SA08-8 attenuated isolate and the C05T virulent isolate revealed two amino acid changes (i.e., _20_S > F and _336_V > I) in the glycoprotein gene; these differences may contribute to the loss of thrip-mediated transmissibility of the SA08-8 strain. However, the mutation responsible for the attenuation effect of SA08-8 remains unknown. Furthermore, while the SA08-8 isolate has not yet been deployed commercially, this is anticipated in the near future.

## 3. Inoculation of Attenuated Viruses

Attenuated isolates are often referred to as ‘plant vaccines’. To administer a true plant vaccine, sap from suitable leaves can be prepared to multiply an attenuated isolate. This sap is then mechanically introduced with carborundum into the cotyledons of ~10-day-old seedlings. Overall, this method is suitable for a small number of seedlings. However, it is not feasible for farmers due to massive differences in cultivation scale.

**Figure 5:**
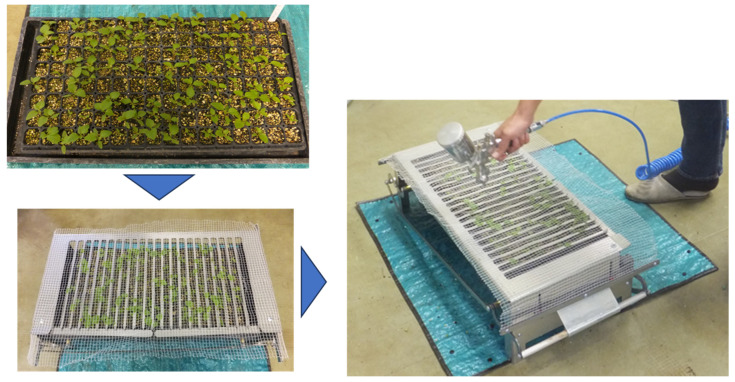
Large-scale inoculation of an attenuated strain using a seedling fixator, a spray gun, and an air compressor. Chinese lantern plants are cultivated using a plug tray (**top left**), and a tray in which seedlings are planted is connected to a seedling fixator (**bottom left**). The cotyledon leaves are supported from below by a comb-shaped structure. Next, a net is placed over the seedlings, and an inoculum is sprayed on them using a spray gun and air compressor (**right**).

To address this issue, large-scale inoculation techniques, such as high-pressure spray guns, have been developed to inoculate tobacco plants with tomato spotted wilt virus [68]. Such large-scale inoculation methods have been further enhanced for practical use, which enables the simultaneous inoculation of thousands of plant seedlings using attenuated strains. For example, the seedling fixator stand (commercially available from Kyoto Biken Laboratories, Inc., Kyoto, Japan) has been developed for large-scale inoculation of attenuated isolates [69]. Its use involves placing a tray of seedlings on the seedling fixator and supporting cotyledon leaves with comb-shaped structures that stick out from below, thereby covering the seedlings with netting. The inoculum is then sprayed onto seedlings using a spray gun and an air compressor (Figure 5). Successful infection of the attenuated isolate was confirmed by RT-PCR using specific primers for the virus. This technique requires at least 0.3 mL of inoculum solution to inoculate one seedling, resulting in over 98% of seedlings being successfully infected with the attenuated isolate. Therefore, this technique is sufficient for the commercial use of the attenuated isolate.

The commercial application of the vaccine is also a crucial task. In Japan, there are two methods for using plant vaccines in the field. The first involves the use of plant vaccines officially registered as biological control agents (i.e., biotic pesticides). Currently, only one product, Cubio ZY-02, is registered in this way. However, its faces minor difficulties, such as the laboriousness of vaccine usage at the point of production (i.e., by farmers themselves). To resolve such problems, the second method involves using seedlings infected with vaccines. For example, Bergearth Co., Ltd. (Kyoto, Japan)., a seedling company, sells cucumber seedlings that are inoculated with multiple vaccines against ZYMV, CMV, and WMV. To date, 8.6 million plants inoculated with Cubio ZY-02 and 1.2 million plants inoculated with both CMV and WMV have been used to manage mosaic diseases in cucumber production areas. In addition, Delmonte Co., Ltd. (Gunma, Japan) sells tomato, green pepper, and cucumber seedlings inoculated with a CMV vaccine.

## 4. Conclusions and Future Prospects

Plant viruses are obligate intracellular parasites that are incapable of replication without a host plant. Currently, no antiviral compounds exist that are capable of treating plants suffering from viral diseases. Conversely, cross-protection has emerged as a promising strategy for global viral disease management. Notably, plant vaccines serve as effective tools for controlling viral diseases in crops that lack native or introduced resistance genes. Despite being a relatively traditional approach, plant vaccines also represent an environmentally sustainable technique.

In commercial agriculture, viruses that elicit either no symptoms or only mild symptoms in plants have been exploited to confer cross-protection against more virulent isolates of the same or closely related viruses, thereby mitigating the impact of crop loss due to viral infection. In the future, the development of multiple plant vaccines that combine several attenuated isolates to generate a vaccine that is effective against multiple viruses is desirable. However, there are several disadvantages related to cross-protection, including: (1) incomplete infection of the protected isolate, which may allow the challenge isolate to invade the plant, (2) breakdown of cross-protection, (3) host-dependence of symptoms, (4) heteroencapsidation, i.e., when genetic material, such as the genome of one virus, is encapsulated within the coat protein of a different virus, (5) mutation of a protective isolate to a severe isolate, (6) hesitance of farmers to intentionally infect crops, and (7) synergistic effects between mild/attenuated viral isolates and unrelated viruses [11]. For example, PepMV shows no defect for cross-protection except for some evidence of synergistic interactions [17].

A study of PMMoV-L3-163 showed that mosaic symptoms occurred in green pepper plants during cultivation, and that this was caused by infection with the wild-type virus [31]. This corresponds to the first category of disadvantage described above. However, the mosaic disease caused by the wild-type virus was only limited in some plants and did not spread to others. This evidence suggests that the attenuated isolate did not infect plants infected by wild-type PMMoV isolates. Hence, evidence suggests that incomplete infection of the protected isolate may allow the challenge isolate to invade the plant. However, the challenge isolate was not itself dispersed in the field, since most of the plants were infected with the attenuated isolate. In case of the other attenuated viruses described in this review, no disadvantageous effects have been reported, although synergistic effects have in general not yet been well analyzed. In addition, plants inoculated with the attenuated virus showed no severe symptoms, but plant growth can be reduced relative to healthy, uninoculated plants. However, growth is generally higher than that found in plants infected with virulent isolates.

The use of attenuated isolates of plant viruses for controlling plant diseases in the field have been reported for viruses such as PepMV, CTV, ToMV, ZYMV and papaya ring spot virus (PRSV) [2,11,13,14,15,16,17,70,71,72]. As described in this paper, the practical implementation of seedlings inoculated with CMV- and WMV-attenuated isolates was first commercialized in 2018 in Japan. Furthermore, the sale of cucumber seedlings inoculated with ZYMV, CMV, and WMV commenced in 2022 to combat major viruses affecting cucumbers in open-field cultivation. Moreover, attenuated isolates of ToMV and TMGMV remain available in the field. However, despite the importance of viruses such as PRSV, attenuated strains have not yet been developed and therefore are not available. In addition, there are many important viruses not described in this paper, including tomato yellow leaf curl virus (TYLCV) and tomato chlorosis virus (ToCV). Although attenuated isolates of these viruses are not currently being produced, they should be developed in the near future. Since TYLCV and ToCV are insect-transmitted viruses, plant inoculation is expected to be difficult. However, mechanical inoculation methods for such viruses have recently also been reported [73,74]. Therefore, we speculate that in the future, additional attenuated viruses will be developed to be deployed as multi-vaccines, and will help to control viral diseases in many plant species.

## Figures and Tables

**Table 1:** Attenuated virus isolates developed in Japan.

Genus	Virus Name (Abbreviation)	Isolate	Method of Attenuated Virus Strain Development	Reference
Closterovirus	Citrus tristeza virus (CTV)	M-16A	Isolation by using host plants Heat treatment	[23]
		HM-55	Isolation by using host plants	[24]
Cucumovirus	Cucumber mosaic virus (CMV)	SR	Isolation by using host plants	[25]
		SRO	Exchange of RNA genome	[26]
		SRK	Exchange of RNA genome	[26]
		K02	Addition of satellite RNA	[27]
		P+fl	Addition of satellite RNA	[28]
Potyvirus	Bean yellow mosaic virus (BYMV)	M11	Heat treatment	[29]
	Lily mottle virus (LMoV)	LMm76-2	Tissue culture	[30]
		LMm93	Tissue culture	[30]
	Potato virus Y (PVY)	N-NA10	Sodium nitrite treatment	[31]
		N-MY10	Mutagenesis-in-tissue culture	[31]
	Soybean mosaic virus (SMV)	–	Heat treatment	[32]
	Sweet potato feathery mottle virus (SPFMV)	10-O	Isolation by using host plants	[34]
	Zucchini yellow mosaic virus (ZYMV)	ZYMV-2002	Heat treatment	[33]
Tobamovirus	Cucumber mild green mosaic virus (CGMMV)	SH33b	Sodium nitrite treatment	[35]
	Pepper mild mottle virus (PMMoV)	Pa18	Heat treatment	[38]
		C-1421	Heat treatment	[39]
		L3-163	Heat treatment	[40]
	Tobacco mosaic virus (TMV)	M_3_	Sodium nitrite treatment	[41]
	Tobacco mild green mosaic virus (TMGMV)	No. 4	Unknown	[42]
	Tomato mosaic virus (ToMV)	L_11_	Heat treatment	[43]
		L_11_A	Isolation by using host plants	[36]
		L_11_A237	Isolation by using host plants	[44]
Tospovirus	Melon yellow spot virus (MYSV)	SA08-8	Heat treatment	[45]

**Table 2:** Cross-protective effects against tobacco mild green mosaic virus (TMGMV) and tomato mosaic virus (ToMV)-virulent isolates of Chinese lantern plants inoculated with attenuated isolates (e.g., TMGMV-No. 4 and ToMV-L_11_A) in the greenhouse and in the field.

Treatment	Percentage of Plants Showing Symptoms	
	Mosaic	Necrosis
Greenhouse		
Uninoculated	100%	0%
TMGMV-No. 4	30%	0%
ToMV-L_11_A	66.7%	0%
TMGMV-No. 4 and ToMV-L_11_A	23.3%	0%
Field		
Uninoculated	0%	16.7%
TMGMV-No. 4	0%	47.4%
ToMV-L_11_A	0%	80.8%
TMGMV-No. 4 and ToMV-L_11_A	0%	0%

Data taken from Yoneda et al. [42].

## Data Availability

No new data were created in this work.
